# Preemptive Analgesic Effect of Intrathecal Applications of Neuroactive Steroids in a Rodent Model of Post-Surgical Pain: Evidence for the Role of T-Type Calcium Channels

**DOI:** 10.3390/cells9122674

**Published:** 2020-12-12

**Authors:** Quy L. Tat, Srdjan M. Joksimovic, Kathiresan Krishnan, Douglas F. Covey, Slobodan M. Todorovic, Vesna Jevtovic-Todorovic

**Affiliations:** 1 Department of Anesthesiology, School of Medicine, University of Colorado Denver, Anschutz Medical Campus, Aurora, CO 80045, USA; quy.tat@cuancshutz.edu (Q.L.T.); srdan.joksimovic@cuanschutz.edu (S.M.J.); SLOBODAN.TODOROVIC@UCDENVER.EDU (S.M.T.); 2 Department of Developmental Biology, Washington University in St. Louis School of Medicine, St. Louis, MO 63110, USA; krishnan@wustl.edu (K.K.); dcovey@wustl.edu (D.F.C.); 3 Taylor Family Institute for Innovative Psychiatric Research, Washington University in St. Louis School of Medicine, St. Louis, MO 63110, USA; 4 Neuroscience Graduate Program, School of Medicine, University of Colorado Denver, Anschutz Medical Campus, Aurora, CO 80045, USA

**Keywords:** incision pain, mechanical hypersensitivity, GABAergic, morphine, voltage-gated calcium channels, adult female rats

## Abstract

Preemptive management of post-incisional pain remains challenging. Here, we examined the role of preemptive use of neuroactive steroids with activity on low-voltage activated T-type Ca^2+^ channels (T-channels) and γ-aminobutyric acid A (GABA_A_) receptors in the development and maintenance of post-incisional pain. We use neuroactive steroids with distinct effects on GABA_A_ receptors and/or T-channels: Alphaxalone (combined GABAergic agent and T-channel inhibitor), ECN (T-channel inhibitor), CDNC24 (GABAergic agent), and compared them with an established analgesic, morphine (an opioid agonist without known effect on either T-channels or GABA_A_ receptors). Adult female rats sustained the skin and muscle incision on the plantar surface of the right paw. We injected the agents of choice intrathecally either before or after the development of post-incisional pain. The pain development was monitored by studying mechanical hypersensitivity. Alphaxalone and ECN, but not morphine, are effective in alleviating mechanical hyperalgesia when administered preemptively whereas morphine provides dose-dependent pain relief only when administered once the pain had developed. CDNC24 on the other hand did not offer any analgesic benefit. Neuroactive steroids that inhibit T-currents—Alphaxalone and ECN—unlike morphine, are effective preemptive analgesics that may offer a promising therapeutic approach to the treatment of post-incisional pain, especially mechanical hypersensitivity.

## 1. Introduction

Most commonly used injectable general anesthetics do not provide a level of analgesia that is required for surgical procedures, necessitating the use of other agents such as opioid analgesics in the perioperative period. Although opioids are very effective in treating the acute pain associated with surgical incisions [1,2,3], they are commonly ineffective and short-lasting when it comes to preemptive post-surgical pain management [4]. Furthermore, it is well known that opioid use is associated with serious side effects, most notably impaired cognitive function, respiratory depression, tolerance and addiction [5]. The opioid abuse epidemic remains a great concern in the United States [6], resulting in more overdose deaths than heroin and cocaine combined [7]. Considering the predictable nature of incisional pain commonly associated with any surgical intervention involving skin or mucosal incision, we believe that new therapeutic modalities that are not habit-forming and are focused on preemptive pain management in the perioperative period are warranted.

It is well known that voltage-gated calcium channels (VGCCs) and low-voltage activated T-type calcium channels (T-channels) in particular, play an important role in controlling the calcium-modulated release of synaptic vesicles from neuronal presynaptic terminals in the spinal dorsal horn [8] and may also directly influence the excitability of sensory neurons in response to noxious stimulation [9]. Hence, we proposed that inhibition of neuronal T-currents in pain pathways may provide effective analgesia. Indeed, our work and the work of others over the past two decades have shown that the blockade of T-currents in nociceptive neurons results in potent peripheral anti-nociceptive effects in a variety of animal pain models [10,11,12,13,14,15,16].

A class of agents that has been of particular interest to us are neuroactive steroids. Our very recently published data provide evidence that the neuroactive steroid with T-channel blocking properties, 3β-OH [(3β,5β,17β)-3-hydroxyandrostane-17-carbonitrile] provides excellent spinally-mediated acute analgesia in an animal model of incisional pain [16]. Combined with our earlier published work this led us to believe that 3β-OH and related steroid molecules may represent a novel class of analgesics with desirable and unique analgesic properties following systemic and intrathecal delivery, as well as delivery at the site of tissue injury [16].

To elevate this work to the next level, we set out to examine the preemptive antinociceptive potential of select neuroactive steroids and to compare it to their acute antinociceptive properties in the setting of incisional pain. We focused on three representative steroid analogs: Alphaxalone [(3α,5α)-3-hydroxypregnane-11,20-dione], ECN [(3β,5α,17β)-17-hydroxyestrane-3-carbonitrile] and CDNC24 [(3α,5α)-3-hydroxy-13,24-cyclo-18,21-dinorchol-22-en-24-ol]. Their acute and preemptive analgesic properties were compared to a well-known analgesic, morphine. The use of Alphaxalone, ECN and CDNC24 enabled us to assess the importance of T-channels and ligand-gated channels (γ-aminobutyric acid - GABA_A_). Specifically, ECN has no effect on GABA_A_ receptors but inhibits T-channels with reasonably high affinity and selectivity [17] whereas CDNC24 effectively potentiates GABA_A_ currents but has no effect on T-currents in sensory neurons [18]. Since Alphaxalone exhibits a combined effect as a GABAergic potentiator and a T-channel blocker [17], the use of Alphaxalone enabled the assessment of both cellular targets simultaneously. The assessment of GABAergic effects vis-à-vis the effects of T-channel modulation was of importance for two main reasons: (1) spinal GABAergic transmission has been shown to modulate nociceptive responses in the acute and chronic pain settings [19] and, (2) the majority of neuroactive steroids are potent GABAergic modulators and their effects on neurosensory processing and neuronal excitability are often mediated by their action at GABA_A_ receptors.

We report that neuroactive steroids with T-channel blocking properties, ECN and Alphaxalone, provide reasonably long-lasting preemptive analgesia in a rat model of surgical incisional pain. This is in contrast to the clinically used analgesic morphine which provides effective and dose-dependent alleviation of acute incisional pain but had no preemptive analgesic properties.

## 2. Materials and Methods

### 2.1. Animals

All our experimental protocols were approved by the Animal Care and Use Committee of the University of Colorado Anschutz Medical Campus and all our animal experiments were performed in accordance to the Guide for the Care and Use of Laboratory Animals (Institute of Laboratory Animal Resources, 1996). The animals were housed on a 12 h light–dark cycle with ad lib food and water supply. We used female rats in our study for two main reasons: (1) females are considered to be more prone to chronic pain states when compared to males in both clinical and preclinical studies [20]; (2) the majority of current pain studies in animals are performed using males [21]. Thus, we believe that the research focus should also be on female pain perception.

Sprague Dawley female rats (6–7 weeks old) were randomly assigned to treatment groups with the experimenter blinded to drug treatment. All efforts were made to reduce animal suffering and the number of animals used while generating the sample size necessary for proper statistical analyses.

### 2.2. Drugs

Alphaxalone [(3α,5α)-3-hydroxypregnane-11,20-dione], ECN [(3β,5α,17β)-17-hydroxyestrane-3-carbonitrile] and CDNC24 [(3α,5α)-3-hydroxy-13,24-cyclo-18,21-dinorchol-22-en-24-ol] are synthetic neurosteroid analogs synthetized using procedures that have been described in our previous publications [11,22,23]. Morphine was purchased through the University of Colorado Hospital Pharmacy (Morphine sulfate inj. USP 4 mg/mL, West Ward). The neuroactive steroids were dissolved in 15% 2-Hydroxypropyl-β-cyclodextrin solution in pH-balanced saline (pH = 7.4 to avoid tissue irritation). In our published studies, using in vitro recordings, we established that the effects of these neuroactive steroids on T-channels and GABA_A_ receptors are within the same concentration range [17,24]. Hence, for that reason, we used the same range of doses of neuroactive steroids for intrathecal injections in our in vivo studies.

### 2.3. Incisional Pain Model

Our standard model for the induction of post-surgical pain using the skin and muscle incision was described previously [15,16,25] as follows: animals were anesthetized with isoflurane (2.5% for induction and for maintenance) and the plantar surface of the right paw was incised longitudinally with a blade No.11; the underlying plantaris muscle was elevated and incised also longitudinally, after which the skin was sutured with 5-0 nylon suture with a FS-2 needle. Animals were allowed to recover in cages, and all experiments were initiated as early as 2 h post-incision.

### 2.4. Intrathecal Injections

Our standard model for intrathecal injections was described previously [15,16] as follows: after briefly anesthetizing animals with isoflurane (2.5% for induction in an induction chamber, and 2.5% for maintenance), the back of each animal was shaved to expose the injection site in the region of L_4_-L_6_ of the spinal column. A 28 G needle was used for the acute i.t. injection. After inserting the needle into the L_4_-L_6_ lumbar region, the experimental compound or vehicle was delivered in a volume of 50 µL, and the animal was left to recover in a cage before initiating experiments.

### 2.5. Mechanical Sensitivity

Our standard method for assessing mechanical sensitivity was described previously [15,16] as follows: we used the electronic Von Frey apparatus (Ugo Basile, Varese, Italy), the apparatus that utilizes a single rigid filament that exerts pressure to the plantar surface of the paw in a range from 0 to 50 g. Animals were placed in plastic enclosures on a wire mesh stand to habituate for 15 min. After habituation, a probe was applied to the plantar surface of the paw through the mesh floor of the stand, and constant force was applied to the mid-plantar area of the paw. As soon as the exerted pressure of the punctate stimulus reaches the maximum force that the animal can endure, immediate brisk paw withdrawal is noticeable, and the force in grams is displayed on the apparatus representing a threshold for paw withdrawal response (PWR). Each paw was tested three times and the average value of threshold PWRs was used in further analysis. Any other voluntary movement of the animal was not considered as a response.

### 2.6. Data Analysis and Statistics

Our standard method for statistical analyses of our data was described previously [15,16] as follows: for all studies rats were age-matched to keep the treatment groups as similar as possible. In order to assure stable recording conditions for the measurements of mechanical sensitivities, we determined baseline values on both paws prior to surgery and again prior to drug application. Each data point from experiments was expressed as mean + or ± SD. All datasets were tested for normality and equal variance (Mauchly’s test of sphericity and Levene’s test of Equality of Equal Variances), and proper statistical analysis of the differences in effects between the treatment and the vehicle groups was performed using two-way RM ANOVA followed by Tukey’s and Sidak’s post hoc tests. To make comparisons with the baseline measurements, we also used one-way RM ANOVA followed by Dunnett’s test. In multigroup studies with parametric variables, post hoc tests (recommended by GraphPad prism) were conducted only if F in ANOVA achieved the necessary level of statistical significance and there was no significant variance inhomogeneity. Significant differences between group means are indicated when *p* < 0.05. GraphPad Prism 8 (GraphPad Software, La Jolla, CA, USA) was used for all statistical analyses.

## 3. Results

**Figure 1:**
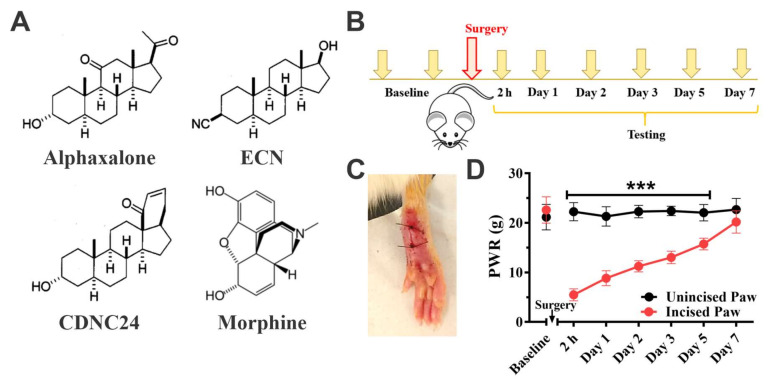
The development of postsurgical mechanical hypersensitivity in rats. (**A**). Chemical structures of compounds investigated in the present study: Alphaxalone, ECN, CDNC24 and Morphine. (**B**). A time-course of the in vivo experiment: after presurgical baseline measurements, the paw withdrawal responses (PWR) to mechanical stimuli were followed for 7 postsurgical days. (**C**). A representative image of incised paw upon completion of the surgery. (**D**). The graph shows the development of mechanical hypersensitivity after incision in rats. Two hours after surgery there was a significant decrease in mechanical sensitivity threshold, confirming pain development, which lasted up to 5 postsurgical days. Each data point is represented as mean ± SD; *n* = 8 rats per data point. *** *p* < 0.001 unincised vs. incised paw, Two-way RM ANOVA followed by Sidak’s post hoc test.

To assess the analgesic properties of neuroactive steroids and their potential role in the management of incisional pain, we focused on three representative steroid analogs: Alphaxalone [(3α,5α)-3-hydroxypregnane-11,20-dione], ECN [(3β,5α,17β)-17-hydroxyestrane-3-carbonitrile] and CDNC24 [(3α,5α)-3-hydroxy-13,24-cyclo-18,21-dinorchol-22-en-24-ol]. Their analgesic properties were compared to a well-known analgesic, morphine (Figure 1A).

In animals that underwent skin incision, we focused on mechanical hypersensitivity as an important feature of incisional pain for two main reasons: it is very commonly observed in a clinical setting of incisional pain [3,26] and as published previously, it is sensitive to pharmacological and biochemical modulation via T-channels [15,16]. As shown in Figure 1B, after determining the baseline mechanical sensitivity threshold, the animals underwent paw skin incision (Figure 1C, as described in Methods) and mechanical sensitivity was assessed daily (up to 7 days). As shown in Figure 1D, when assessed two hours post-incision, there was a significant mechanical hypersensitivity, hence confirming acute pain development. When assessed daily, the mechanical hypersensitivity of the incised paw remained significant up to 5 post-surgical days when compared to unincised (contralateral) paw (factor treatment: F_1,14_ = 340.7, *p* < 0.001; interaction: F_5,70_ = 57.45, *p* < 0.001; post hoc: *** *p* < 0.001). By Day 7, the mechanical threshold was not significantly different from the pre-incision baseline (*p* = 0.068) or unincised paw (*p* = 0.234).

**Figure 2:**
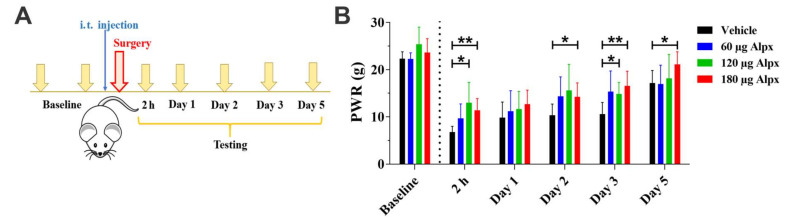
The preemptive administration of Alphaxalone alleviates postincisional mechanical hypersensitivity in rats. (**A**). A time-course showing presurgical baseline measurements, the timing of intrathecal (i.t.) Alphaxalone (Alpx) injection and postsurgical testing time points. (**B**). The preemptive i.t. application of Alpx alleviated postincisional mechanical hypersensitivity in rats in a dose-dependent manner (*n* = 9 for Vehicle, 60 µg and 120 µg Alpx groups; *n* = 8 for the 180 µg Alpx group). Each data point is represented as mean + SD. * *p* < 0.05 and ** *p* < 0.01 vs. Vehicle group, Two-way RM ANOVA followed by Tukey’s post hoc test.

**Figure 3:**
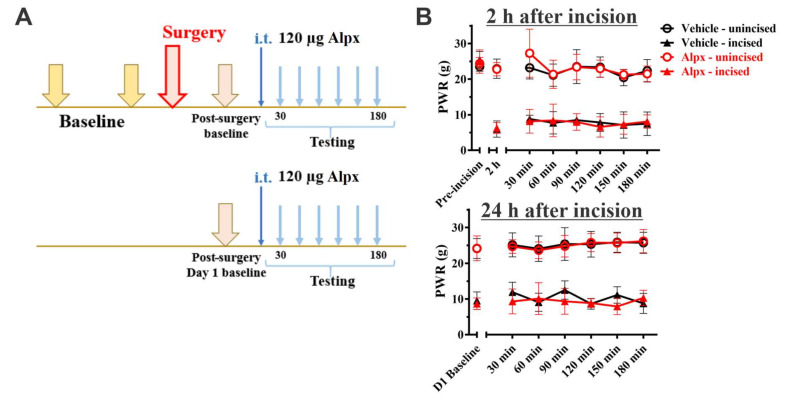
Repeated administration of Alphaxalone does not affect postincisional mechanical hypersensitivity in rats. (**A**). A time-course showing baseline measurements to mechanical stimuli two days before incision, two hours and 24 h after incision. The paw withdrawal responses were measured every thirty minutes after i.t. injection of 120 µg Alpx. (**B**). Repeated administration of 120 µg Alpx at 2 h (top; *n* = 5 per group) or 24 h after incision (bottom; *n* = 6 per group) does not alleviate mechanical hypersensitivity in rats. Each data point is represented as mean ± SD. Data were analyzed using Two-way RM ANOVA.

**Figure 4:**
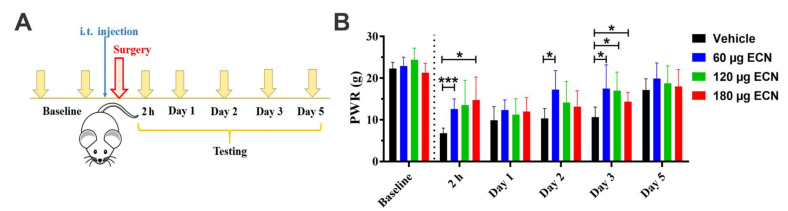
The preemptive administration of ECN alleviates postincisional mechanical hypersensitivity in rats. (**A**). A time-course showing presurgical baseline measurements, the timing of intrathecal (i.t.) ECN injection and post-surgical testing time points. (**B**). The preemptive i.t. application of ECN alleviated post-incisional mechanical hypersensitivity in rats in a dose-dependent manner (*n* = 9 for Vehicle; *n* = 8 for 60 µg, *n* = 7 for 120 µg and *n* = 8 for 180 µg ECN groups). Each data point is represented as mean + SD. * *p* < 0.05 and *** *p* < 0.001 vs. Vehicle group, Two-way RM ANOVA followed by Tukey’s post hoc test.

**Figure 5:**
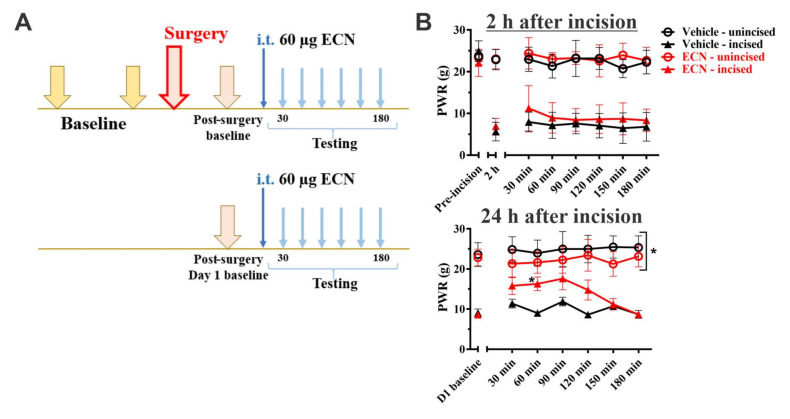
Repeated administration of ECN alleviates post-incisional mechanical hypersensitivity in rats. (**A**). A time-course showing baseline measurements to mechanical stimuli two days before incision, two hours and 24 h after incision. The paw withdrawal responses were measured every thirty minutes after i.t. injection of 60 µg ECN. (**B**). A single i.t. injection of 60 µg ECN two hours after incision does not affect mechanical hypersensitivity (top; *n* = 6 for Vehicle and *n* = 9 for ECN groups). Second dose of ECN 24 h after incision significantly increases the mechanical sensitivity threshold in the incised paw (bottom; *n* = 7 for Vehicle and *n* = 8 for ECN groups). Each data point is represented as mean ± SD. * *p* < 0.05 vs. Vehicle group, Two-way RM ANOVA followed by Sidak’s post hoc test.

**Figure 6:**
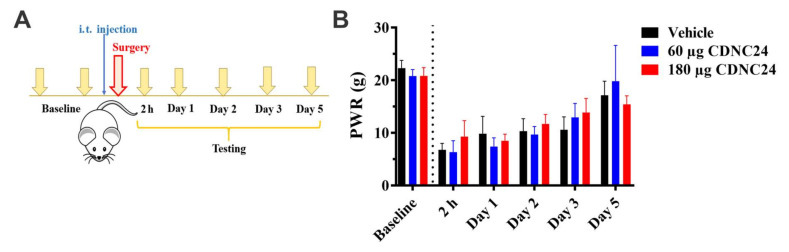
The preemptive administration of CDNC24 does not alleviate post-incisional mechanical hypersensitivity in rats. (**A**). A time-course showing pre-surgical baseline measurements, the timing of intrathecal (i.t.) CDNC24 injection and post-surgical testing time points. (**B**). The preemptive i.t. application of CDNC24 did not affect post-incisional mechanical hypersensitivity in rats (*n* = 9 for Vehicle; *n* = 7 for 60 µg and 180 µg CDNC24 groups). Each data point is represented as mean + SD. Data were analyzed using Two-way ANOVA.

Once we established the basic pattern and severity of mechanical hypersensitivity in our animal model of incisional pain, we set out to study the role of Alphaxalone (Figure 2 and Figure 3), ECN (Figure 4 and Figure 5) and CDNC24 (Figure 6) in managing the incisional pain when applied preemptively or repeatedly.

As stated earlier, Alphaxalone is a potent GABAergic potentiator and an inhibitor of T-channels in sensory neurons [17]. The preemptive intrathecal (i.t.) application of either one of three doses of Alphaxalone (60, 120 or 180 μg) occurred immediately before skin incision, whereas the testing was initiated 2 h post-incision and was carried out daily up to five days (Figure 2A). As could be seen in Figure 2B, there was a dose-dependent and statistically significant reduction in mechanical hypersensitivity, as compared to the vehicle group (factor treatment: F_3,31_ = 6.03, *p* = 0.002; interaction not significant; post hoc: * *p* < 0.05, ** *p* < 0.01). The dose-dependent effect appears to be significant in terms of the reduction in mechanical hypersensitivity and in terms of the duration of the analgesic effect. Although we detected a significant effect within the first two hours post-incision, it was not until Day 2 that we observed a steady reduction in mechanical hypersensitivity post-Alphaxalone injection when compared to control animals. Furthermore, at the highest dose (180 μg), a single preemptive i.t. injection resulted in a long-lasting relief (as detected on Day 5) leading to “normalization” of mechanical response when compared to the baseline (factor treatment: F_5,40_ = 64.30, *p* < 0.001; post hoc: *p* = 0.262). It is noteworthy that this dosing regimen had no effect on mechanical sensitivity at any dose of Alphaxalone in the unincised paw when compared to the vehicle group (factor treatment: F_3,31_ = 1.53, *p* = 0.226; interaction not significant; data not shown).

To assess the effect of repeated Alphaxalone administration on mechanical hypersensitivity during the acute phase of incisional pain, we administered Alphaxalone two hours and 24 h post-incision and mechanical hypersensitivity was measured in 30 min-intervals post-Alphaxalone injection after we confirmed the mechanical hypersensitivity when compared to the baseline (Figure 3A). Since we did not detect a significant difference in PWRs between preemptively administered doses of 120 µg and 180 µg Alphaxalone at 2 hrs post-surgery (Figure 2B), we used a lower dose considering the need for repeated injections in post-incisional application paradigm. Unlike the findings presented in Figure 2B, 120 μg of Alphaxalone, given at 2 h post-incision once mechanical hypersensitivity had developed, did not have any effect on mechanical thresholds when compared to vehicle or post-incision baseline (Figure 3B, top), as determined up to 180 min post-injection (factor treatment: F_1,8_ = 0.02, *p* = 0.902; interaction not significant). To examine further the effect of Alphaxalone during the acute phase of post-incisional pain, we repeated the intrathecal Alphaxalone injection at 120 μg 24 h after incision (Figure 3B, bottom). When measured in 30 min-intervals (up to 180 min), there was again no effect of Alphaxalone on mechanical hypersensitivity in the incised paw (factor treatment: F_1,10_ = 2.09, *p* = 0.179; interaction not significant). Furthermore, this dosing regimen had no effect on mechanical sensitivity in the unincised paw when compared to the baseline or vehicle at either 2 h (Figure 3B, top) or 24 h (Figure 3B, bottom).

To further assess the analgesic potential of neuroactive steroids, we next focused on ECN, a steroid analog that has no effect on GABA_A_ receptors but inhibits T-channels with reasonably high affinity and selectivity [17]. The timeline presented in Figure 4A shows that the preemptive i.t. application of each one of three doses of ECN (60, 120 or 180 μg) occurred immediately before skin incision, whereas the testing was initiated 2 h post-incision and was carried out daily up to five days. As shown in Figure 4B, there was a dose-dependent and statistically significant reduction in mechanical hypersensitivity (factor treatment: F_3,28_ = 7.13, *p* = 0.001; interaction not significant; post hoc: * *p* < 0.05, *** *p* < 0.001 compared to the vehicle group). Based on our findings, we made two important observations. First, a dose-dependent effect appears to be pronounced already two hours post-incision and second, ECN at the lowest dose (60 μg) is at least as effective as two higher doses (120 and 180 μg) in alleviating mechanical hypersensitivity up to three days after incision. It is noteworthy that this dosing regimen basically had no effect on mechanical sensitivity in the unincised (contralateral) paw (factor treatment: F_3,28_ = 1.45, *p* = 0.251; interaction not significant; data not shown).

To assess the effect of repeated ECN administration on mechanical hypersensitivity during the acute phase of incisional pain, we administered 60 μg of ECN intrathecally two hours and 24 h post-incision and, immediately after, we assessed the mechanical hypersensitivity in 30 min-intervals post-ECN injection (Figure 5A). Unlike Alphaxalone (Figure 3), ECN at 60 μg given once mechanical hypersensitivity had developed provided some alleviation of mechanical hypersensitivity during the acute phase of post-incisional pain. Specifically, although we noted no effect on mechanical thresholds when compared to vehicle 2 h after incision (Figure 5B, top; factor treatment: F_1,13_ = 1.73, *p* = 0.211; interaction not significant), we detected a significant alleviation after the second dose that was given 24 h after incision (Figure 5B bottom; factor treatment: F_1,13_ = 5.27, *p* = 0.039; interaction: F_5,65_ = 3.29, *p* = 0.010). Namely, when the second ECN 60 μg injection was given 24 h after incision, we noted a significant alleviation of mechanical hypersensitivity 60 min post-treatment compared to the vehicle group (* *p*<0.05). We detected no change in PWRs at 150 min after injection. This dosing regimen had only a minimal effect on mechanical sensitivity in the unincised paw when compared to the baseline or vehicle recordings at two hours (a small but significant change was noted at 24 hrs post-incision as compared to the vehicle group—factor treatment: F_1,13_ = 6.28, *p* = 0.026; interaction not significant).

A third neuroactive steroid, CDNC24, effectively potentiates GABA_A_ currents but has no effect on T-currents in sensory neurons [18]. CDNC24 was injected preemptively at either one of two doses (60 or 180 μg, i.t.) immediately before skin incision (Figure 6A) whereas the testing was initiated two hours post-incision and daily for up to five days (Figure 6A). We observed no effect on mechanical hypersensitivity at either dose when compared to the vehicle group in the incised paw (Figure 6B; factor treatment: F_2,20_ = 7.28, *p* = 0.495; interaction: F_8,80_ = 3.02, *p* = 0.005). It is noteworthy that this dosing regimen had no effect on mechanical sensitivity at either dose of CDNC24 in the unincised paw, except for a very transient effect on Day 3 at 60 μg (factor treatment: F_2,20_ = 0.391, *p* = 0.681; interaction: F_8,80_ = 3.00, *p* = 0.005; post hoc: *p* = 0.046; data not shown).

**Figure 7:**
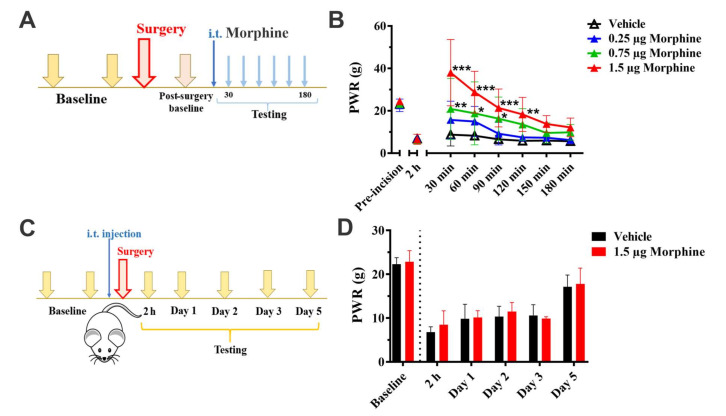
The preemptive administration of morphine does not alleviate post-incisional mechanical hypersensitivity in rats. (**A**). A time-course showing baseline measurements to mechanical stimuli two days before incision, two hours and 24 h after incision. The paw withdrawal responses were measured every thirty minutes after intrathecal (i.t.) injection of morphine (0.25 µg, 0.75 µg or 1.5 µg). (**B**). Morphine significantly increases the mechanical sensitivity threshold in the incised paw in a dose-dependent manner (*n* = 8 for Vehicle; *n* = 7 for 0.25 µg, *n* = 9 for 0.75 µg and *n* = 8 for 1.5 µg morphine groups). Each data point is represented as mean ± SD. * *p* < 0.05, ** *p* < 0.01 and *** *p* < 0.001 vs. Vehicle group, Two-way ANOVA followed by Tukey’s post hoc test. (**C**). A time-course showing pre-surgical baseline measurements, the timing of i.t. morphine injection and post-surgical testing time points. (**D**). Preemptive treatment of 1.5 µg morphine does not reduce mechanical hypersensitivity after surgery (*n* = 9 for Vehicle and *n* = 5 for 1.5 µg morphine group). Each data point is represented as mean + SD. Data were analyzed using Two-way ANOVA.

As a point of comparison, we used a well-established and clinically-used analgesic without any known effects on either T-type or GABA_A_ currents, morphine. First, we tested three doses of intrathecally administered morphine (0.25, 0.75 and 1.5 μg) in 30 min-intervals after we established the mechanical hypersensitivity in the incised paw (Figure 7A). As expected, morphine produced a clear dose-dependent and statistically significant reduction in mechanical hypersensitivity (Figure 7B; * *p* < 0.05, ** *p* < 0.01, *** *p* < 0.001 compared to the vehicle group). The highest dose and the one shown to be most effective in providing analgesia when morphine was given post-skin incision (1.5 μg) was used in the ensuing preemptive experiment. As shown in Figure 7C, intrathecal application of morphine at 1.5 μg occurred preemptively immediately before skin incision whereas the testing was initiated two hours post-incision and was carried out daily up to five days. We discovered that preemptively given morphine, unlike Alphaxalone (Figure 2) or ECN (Figure 4), had no effect on mechanical hypersensitivity when compared to the vehicle group (Figure 7D; factor treatment: F_1,12_ = 0.77, *p* = 0.396; interaction not significant). It is noteworthy that preemptively administered morphine had no effect on mechanical sensitivity in the unincised paw (factor treatment: F_1,12_ = 2.21, *p* = 0.163; interaction not significant; data not shown).

## 4. Discussion

In this study, we show that neuroactive steroids that inhibit T-currents like Alphaxalone and ECN are effective preemptive analgesics that may offer a promising therapeutic approach to the treatment of post-incisional pain. In contrast, a widely used analgesic, morphine despite being very effective in treating acute incisional pain once developed, had no preemptive analgesic effect. Since post-incisional pain is a common occurrence after any surgical intervention that involves skin or mucosa incision and as such it is highly predictable, being able to prevent or lessen its burden and potentially decrease the opioid requirements post-operatively would be of great benefit to our surgical patients.

The neuroactive steroids are potent modulators of neuronal activity in the peripheral and central nervous system [27]. Their effects on neurosensory processing and neuronal excitability are primarily mediated by actions at various ligand-gated ion channels, with much attention focused on the modulation of γ-aminobutyric acid (GABA_A_) receptors [28]. However, VGCCs are rapidly emerging as an important therapeutic target of a variety of neuroactive steroids. Neuroactive steroids modulate several subtypes of VGCCs expressed on neurons along the pain pathway and hence are crucial not only in shaping action potentials, but also in controlling cellular excitability and synaptic neurotransmission in the pain pathway [15,29]. Of particular interest to our study is the effect of neuroactive steroids on T-channels since they have been shown to support nociceptive sensory transmission. Many recent studies have shown that T-channels contribute to nociceptive sensitization [8,10,12,30] and that T-channel blockers could be a promising therapeutic approach to acute and chronic pain treatment including surgical incision [15,16,18,31,32].

Since neuroactive steroids used in this study and previously published ones [18,33] have been shown to be formidable analgesics for the treatment of both acute and chronic pain, the question became whether this is perhaps a universal property of neuroactive analogs with a core pregnane ring system or whether such neurosteroids are cellular target-specific. Our work presented herein would suggest that their analgesic properties are specific to a cellular target, T-channels in particular. For example, ECN, a neuroactive steroid with a 5α configuration at the steroid A,B ring fusion which we show herein to be a good preemptive analgesic is a potent voltage-dependent blocker of T-channels in rat DRG neurons (IC_50_ of 300 nM, maximal block 40% current) while being a weak blocker of recombinant Ca_V_2.3 currents (IC_50_ 16 μM) [34], with very little effect on voltage-gated Na^+^, K^+^, N- and L-type HVA Ca^2+^ channels, glutamate and GABA-gated channels [17]. Along the same lines, several synthetic 5β-reduced steroid analogues that potently and completely inhibit T-currents in DRG cells also exhibit potent local analgesic effects in vivo [11]. One of the most potent and efficacious steroid analogues in this group, 3β-OH ((3β,5β,17β)-3-hydroxyandrostane-17-carbonitrile) is a voltage-dependent and selective blocker of T-currents in acutely dissociated DRG cells (IC_50_ 3 μM, 100% maximal block).

Notably, previous studies have shown that neither ECN [17] nor 3β-OH [23] has any direct effect on GABA_A_ currents which would suggest that GABAergic potentiation could not explain the potent analgesic effects of pregnane-based neurosteroid analogs. Indeed, we show that CDNC24 which has no effect on T-currents but is a potent potentiator of GABA_A_ currents [18] and anesthetic in tadpoles [35] has no analgesic properties in the incisional pain model thus suggesting that the observed analgesic effect of Alphaxalone was most likely due to its T-channel blocking properties [17] rather than its GABAergic properties [28] or pregnane-based ring structure [36].

The mechanisms of preemptive analgesia as well as the clinical effectiveness of current approaches remain a puzzle. The recent review [37] that summarized years of clinical practice focused on preemptive analgesia for thoracic surgeries questions whether it is even “an obtainable goal”. Despite the utilization of a variety of regional and neuroaxial analgesic techniques before surgery, post-operative pain remains a major stumbling block even when a variety of analgesics—opioids, local anesthetics and non-steroidal anti-inflammatory medications are used [38]. Along those lines, previously published animal work suggests that pre-incisional administration of the analgesic ketamine, a potent antagonist of the NMDA subtype of glutamate receptors results in pain relief that is not more effective or longer lasting than when ketamine is administered post-incisionally thus questioning the benefits of a pre-emptive pain management approach [39]. Our work with morphine when compared to ECN and Alphaxalone we present herein suggests that the common notion regarding the effectiveness of currently available preemptive modalities is most likely related to the cellular target that is being modulated. In other words, the cellular receptor systems being targeted by currently available analgesics may not be promising ones when it comes to the effectiveness of preemptive analgesia. We believe that our findings regarding preemptive blockade of T-channels that leads to longer-lasting relief of post-incisional pain are due to the prominent role that T-channels play not only in shaping action potentials, but also in controlling cellular excitability and synaptic transmission within the neurons along the pain pathway. Since T-channels are known to significantly contribute to nociceptive sensitization [8,10,12,15,16,30], timely quieting of nociceptive hypersensitivity may be key to successful preemptive pain management.

Our findings confirm previously published findings regarding questionable preemptive analgesic benefits of morphine [40,41]. Considering the significant side effects of opioids that could be life-threatening or life-altering, the multimodal approach which relies on preemptive administration of T-channel blocking neuroactive steroids followed by their use post-operatively may offer a workable and safe alternative to opioids [42,43]. Although not tested in our current study, the possibility that preemptive and postoperative use of neuroactive steroids such as ECN, Alphaxalone and 3β-OH may decrease the opioid requirements postoperatively or eliminate them altogether is an appealing one and worthy of being confirmed in future animal studies.

In this study we focused our attention on controlling the development of mechanical hypersensitivity post-incision since our previously published work using the same model has shown that T-channel blockade appears to control the development of mechanical hypersensitivity to a higher degree than thermal hypersensitivity [15,16]. Our findings are not unique in the sense that based on other reports, different pain modalities likely rely on different cellular mechanisms. For example, antagonists of acid-sensing ion channels provide questionable relief from heat hyperalgesia after carrageenan injection into the muscle, but are effective in ameliorating mechanical hyperalgesia [44]. On the other hand, in vivo knockdown of acid-sensing ion channels (ASIC3 in particular) leads to a complete remission from heat hyperalgesia after Complete Freund’s Adjuvant (CFA) injection [45] suggesting that different pain etiologies result in pain modalities with unique pharmacological requirements. Hence, it should be of no surprise that the treatment of post-injury pain is a very complex phenomenon in the clinical setting and as such, it is clear why “one size does not fit all”.

## 5. Conclusions

Preemptive analgesia remains a major puzzle in the clinical setting of tissue injury since it does not respond to conventional opioid treatment. Hence there is a keen interest focused on finding ways to provide reliable relief especially since post-injury pain is highly predictable. Considering the complex nature of this phenomenon, we propose that a promising approach should rely on the timely blockade of T-channels with novel neuroactive steroids since they seem to provide lasting relief without habit-forming potential.
